# Research on orchard navigation method based on fusion of 3D SLAM and point cloud positioning

**DOI:** 10.3389/fpls.2023.1207742

**Published:** 2023-06-26

**Authors:** Ye Xia, Xiaohui Lei, Jian Pan, LuWei Chen, Zhen Zhang, Xiaolan Lyu

**Affiliations:** ^1^ Institute of Agricultural Facilities and Equipment, Jiangsu Academy of Agricultural Sciences, Nanjing, China; ^2^ Key Laboratory of Modern Horticultural Equipment, Ministry of Agriculture and Rural Affairs, Nanjing, China; ^3^ School of Agricultural Engineering, Jiangsu University, Zhenjiang, China

**Keywords:** orchard, robot, autonomous navigation, vector map, LiDAR, SLAM

## Abstract

Accurate navigation is crucial in the construction of intelligent orchards, and the need for vehicle navigation accuracy becomes even more important as production is refined. However, traditional navigation methods based on global navigation satellite system (GNSS) and 2D light detection and ranging (LiDAR) can be unreliable in complex scenarios with little sensory information due to tree canopy occlusion. To solve these issues, this paper proposes a 3D LiDAR-based navigation method for trellis orchards. With the use of 3D LiDAR with a 3D simultaneous localization and mapping (SLAM) algorithm, orchard point cloud information is collected and filtered using the Point Cloud Library (PCL) to extract trellis point clouds as matching targets. In terms of positioning, the real-time position is determined through a reliable method of fusing multiple sensors for positioning, which involves transforming the real-time kinematics (RTK) information into the initial position and doing a normal distribution transformation between the current frame point cloud and the scaffold reference point cloud to match the point cloud position. For path planning, the required vector map is manually planned in the orchard point cloud to specify the path of the roadway, and finally, navigation is achieved through pure path tracking. Field tests have shown that the accuracy of the normal distributions transform (NDT) SLAM method can reach 5 cm in each rank with a coefficient of variation that is less than 2%. Additionally, the navigation system has a high positioning heading accuracy with a deviation within 1° and a standard deviation of less than 0.6° when moving along the path point cloud at a speed of 1.0 m/s in a Y-trellis pear orchard. The lateral positioning deviation was also controlled within 5 cm with a standard deviation of less than 2 cm. This navigation system has a high level of accuracy and can be customized to specific tasks, making it widely applicable in trellis orchards with autonomous navigation pesticide sprayers.

## 1 Introduction

As a labor-intensive industry (Lyu et al., 2020), fruit production relies heavily on manual labor for its production process. With today’s increasing labor costs and population pressure, traditional production methods are unsustainable. In order to overcome these problems, many studies have been devoted to the use of orchard robots to replace manual labor, and devices such as automated orchard picking robots (Wang X. et al., 2023; Wang Y. et al., 2023) and mobile orchard applications and fertilization robots (Yayan et al., 2015; Gao et al., 2020) have emerged. How to achieve accurate and efficient autonomous navigation in a complex orchard environment by determining its own position through various sensors is the focus of all orchard operation robots. Early autonomous navigation devices in orchards were mainly based on physical tracks (Keicher and Seufert, 2000; Yayan et al., 2015) with global navigation satellite system (GNSS) (Ryu et al., 2016; Yin et al., 2018). Bakker et al. (2011) developed a real-time kinematics–Differential Global Positioning System (RTK-DGPS) automatic navigation platform for sugar beet fields, which can achieve centimeter-level navigation accuracy. Bin et al. (2017) designed an orchard sprayer based on the BeiDou satellite navigation system, which was tested to have an average positioning accuracy of 0.03 m at 2 km/h operating conditions. Some orchards may pose a challenge for GNSS-based navigation devices as agricultural robots often work under the plant canopy. This can result in satellite signals being blocked and not reaching GNSS receivers, as highlighted by Li et al. (2009) and Niewola (2020). Therefore, many researchers have started to replace GNSS as the main sensor for navigation with light detection and ranging (LiDAR) for orchard navigation tasks. Bayar et al. (2015) completed the localization and steering control of orchard vehicles between rows of fruit trees using LiDAR, which is applicable to most orchards and has reliable localization accuracy. Zhang et al. (2020) obtained the trunk position by using 2D LiDAR acquisition and filtering, then fitted the navigation path using least squares, and finally used an improved tracking controller to achieve autonomous navigation. Li et al. (2022) constructed a raster map for the jujube orchard by LiDAR with the 2D simultaneous localization and mapping (SLAM) method and used DWA+A* to plan the path for navigation. Wang et al. (2022) constructed a map by 3D LiDAR for environment sensing while fusing multi-source information with millimeter-wave radar for obstacle avoidance. The navigation test shows its positioning accuracy within 15 cm.

Computer vision techniques also play an important role in the navigation of agricultural machinery. Visual navigation usually uses monocular and binocular cameras with the Hough transform (Hough and Paul, 1962; Winterhalter et al., 2018), least squares (Cui et al., 2015; Mao et al., 2019), and other methods to extract paths. With the rise of image deep learning processing techniques in recent years, many researchers have started to use deep learning processing orchard environment information and fit navigation lines to control vehicles traveling through the orchard. Cao et al. (2022) segmented farm crops based on residual networks and fitted the navigation paths by the least squares method, and their crop row detection accuracy reached 90.9%. Yang et al. (2022) fitted the navigation paths of orchard hard surfaces by training a semantic segmentation network based on SegNet (Badrinarayanan et al., 2017) and UNet (Ronneberger et al., 2015), and their path recognition rate reached 92%. Opiyo et al. (2021) obtained the navigation paths by extracting the texture features of fruit trees and roads, and their lateral accuracy is at the centimeter level.

With the standardized orchard development, trellis orchards are more adapted to mechanized operation. Due to the interference of the trellis and dense canopy, the GNSS signal is unstable, and the positioning method relying on GNSS alone is not reliable. The navigation method of extracting trunk position information by laser filtering is prone to failure due to missing path information at orchard corners. The navigation method of constructing raster maps by LiDAR tends to lose its own position due to the similarity of information between orchard rows. The visual extraction filter is too dependent on the light conditions, which is unsuitable for some working conditions that require night operation. In addition, today’s orchard navigation tasks are more focused on the synergy of multiple tasks, and more intuitive orchard maps are needed to arrange various tasks. Traditional navigation methods mainly construct two-dimensional maps with little information and poor readability. It cannot reflect more effective orchard point cloud information, so a more intuitive way to construct orchard maps is urgently needed to match various mechanical operations. To address these problems, this paper proposes a trellis orchard navigation method based on 3D SLAM technology to construct maps and fuse RTK and LiDAR sensors for redundant positioning. The method uses normal distributions transform (NDT) mapping (Biber and Straßer, 2003) to construct 3D point clouds of orchards and extracts trellis feature points as reference point clouds by straight pass filtering, voxel filtering, outlier filtering, etc. The coordinates provided by RTK are transformed into the initial values of the position in real time and introduced into NDT matching for point cloud matching. Finally, the vector map (Darweesh et al., 2017) of the orchard is constructed in the form of manual planning by marking the paths in the constructed 3D point cloud map of the orchard to adapt to the navigation of various orchards with different scales and usage requirements.

## 2 Materials and methods

### 2.1 Orchard conditions

**Figure 1:**
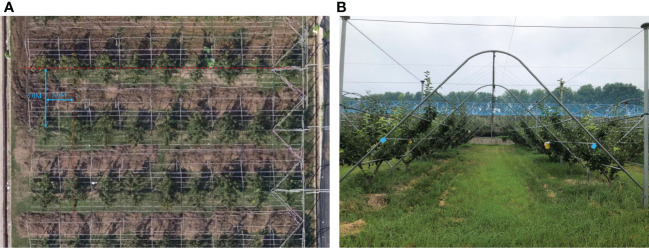
Test fields. **(A)** Aerial view of the orchard. **(B)** Y-trellis for orchard.

The trial site was located in the pear orchard of Jiangsu Academy of Agricultural Sciences, which was constructed using a standard Y-trellis with fruit trees spaced 6.0 m apart in rows, 3.0 m apart in columns, and an average height of 3 m. The trial was conducted in November 2022. **Figures 1A, B** show the aerial view of the orchard and the trellis, respectively.

### 2.2 Hardware system

**Figure 2:**
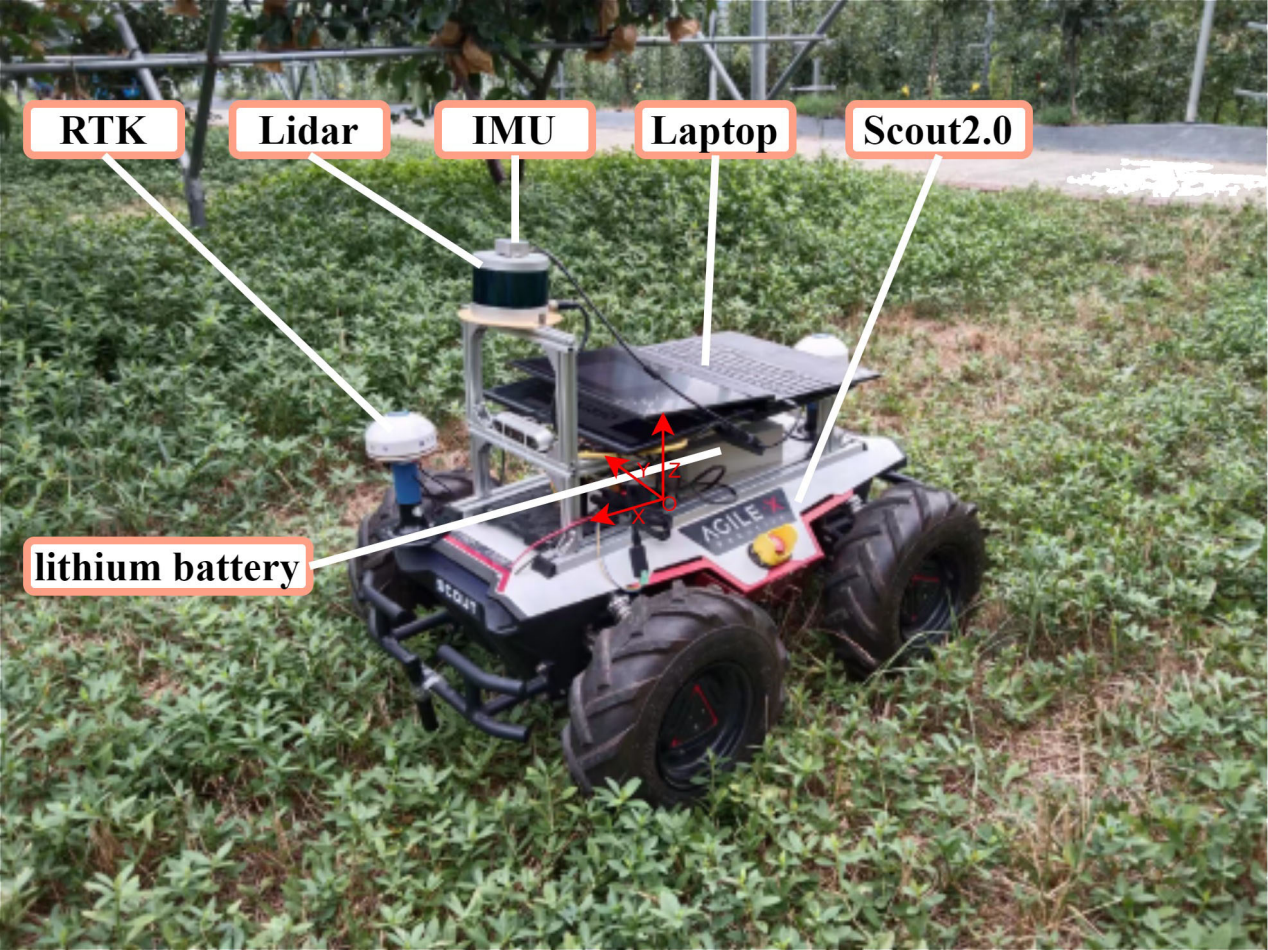
Photograph of the mobile platform.

The study uses Songling Scout 2.0 robot as the motion chassis, which has four-wheel drive capability and uses differential speed for steering. For robot control, the target line and angular speed were received from the host computer via CAN bus and output to the motor for bottom control via STM32. The main technical parameters of the robot chassis are shown in **Table 1**. The navigation system was equipped with a 16-line LiDAR (C16, Leishen Intelligent System Co., Ltd., Shenzhen, China) and a nine-axis inertial measurement unit (IMU) (HFI-A9, HandsFree, Shenzhen, China). It was mounted on a metal bracket at 0.2 m in the X-axis direction and 0.25 m in the Z-axis direction with the center of the robot chassis as the origin of the coordinate system. GNSS (T3-B, QFRTK, Shenzhen, China) equipment was magnetically mounted to the front and rear center of the vehicle chassis to provide differential position information and record real-time trajectories. The laptop was used as a control terminal for various sensors. The laptop consisted of an Intel Core i7-12700 h CPU with 16 GB of DDR5 RAM. Ubuntu Linux 18.04LTS was installed on the computer, together with the Robot Operating System (ROS). The Laptop communicates with the STM32 in the chassis via usb2can with CAN signal output speed information. **Figure 2** shows the complete system hardware platform.

**Table 1:** Main parameters of Scout 2.0 robot.

Name	Value
Dimensions (L × W × H), m	0.93 × 0.7 × 0.35
Wheelbase, mm	498
Tread, mm Top speed, m/s	582 1.5
Motor rated voltage Minimum ground clearance, mm Maximum grade, ° Maximum load, kg	24V DC 135 30 50

### 2.3 3D SLAM-based map construction method

NDT was proposed by Biber and Straßer, 2003. The idea of NDT can be summarized as filtering the input point cloud in each frame and then stitching it with the previous frame by matching the computed bit pose to build a map. NDT mapping first divides the single-frame point cloud scanned by 3D LiDAR into cubes with certain voxel dimensions to reduce the data complexity and introduce an initial set of positional parameters. Formula 1 represents the point cloud intensity of each cube. Then, the probability density is calculated from Formula 2 for all point clouds within the cube. Then, a set of discrete point clouds is expressed directly and approximately in the form of probability density functions, which are smooth and continuously derivable. Each probability density function can be considered as an approximation of a local surface, which not only describes the location of the surface in space but also contains information about the orientation and smoothness of the surface.


(1) $$\text{Σ}=\frac{1}{m}\sum_{k=1}^{m}\left({\vec{y}}_{k}-\vec{\mu }\right){\left({\vec{y}}_{k}-\vec{\mu }\right)}^{T}$$

(2) $$f(\vec{x})=\frac{1}{{\left(2\pi \right)}^{\frac{3}{2}}\sqrt{\left|\text{Σ}\right|}}e^{-\frac{{\left(\vec{x}-\vec{\mu }\right)}^{T}{\text{Σ}}^{-1}(\vec{x}-\vec{\mu })}{2}}$$

where ${\vec{y}}_{k}$ *k*=1,…,*m* are the positions of the reference scan points contained in the cube.

After the transformation of the input point cloud in one frame was completed in this way, the NDT SLAM will update the point cloud in the global map. According to Formula 3, when the point cloud information was input in the next frame, for the point cloud $X=\{{\vec{X}}_{1},\ldots ,{\vec{X}}_{n}\}$ , there exists a set of transformation matrices $T\left(\vec{p},\vec{x}\right)$ . For a given probability density function of the scanned point cloud, the bit pose $\vec{p}$ that makes the best match between the two frames was the solution of the maximum likelihood function, which was also the minimal value of its negative logarithm, as shown in Formula 4.


(3) $$\Psi =\prod_{k=1}^{n}p(T(\vec{p},{\vec{x}}_{k}))$$

(4) $$-\log\text{Ψ}=-\sum_{k=1}^{n}\log\left(p\left(T\left(\vec{p},{\vec{x}}_{k}\right)\right)\right)$$

**Figure 3:**
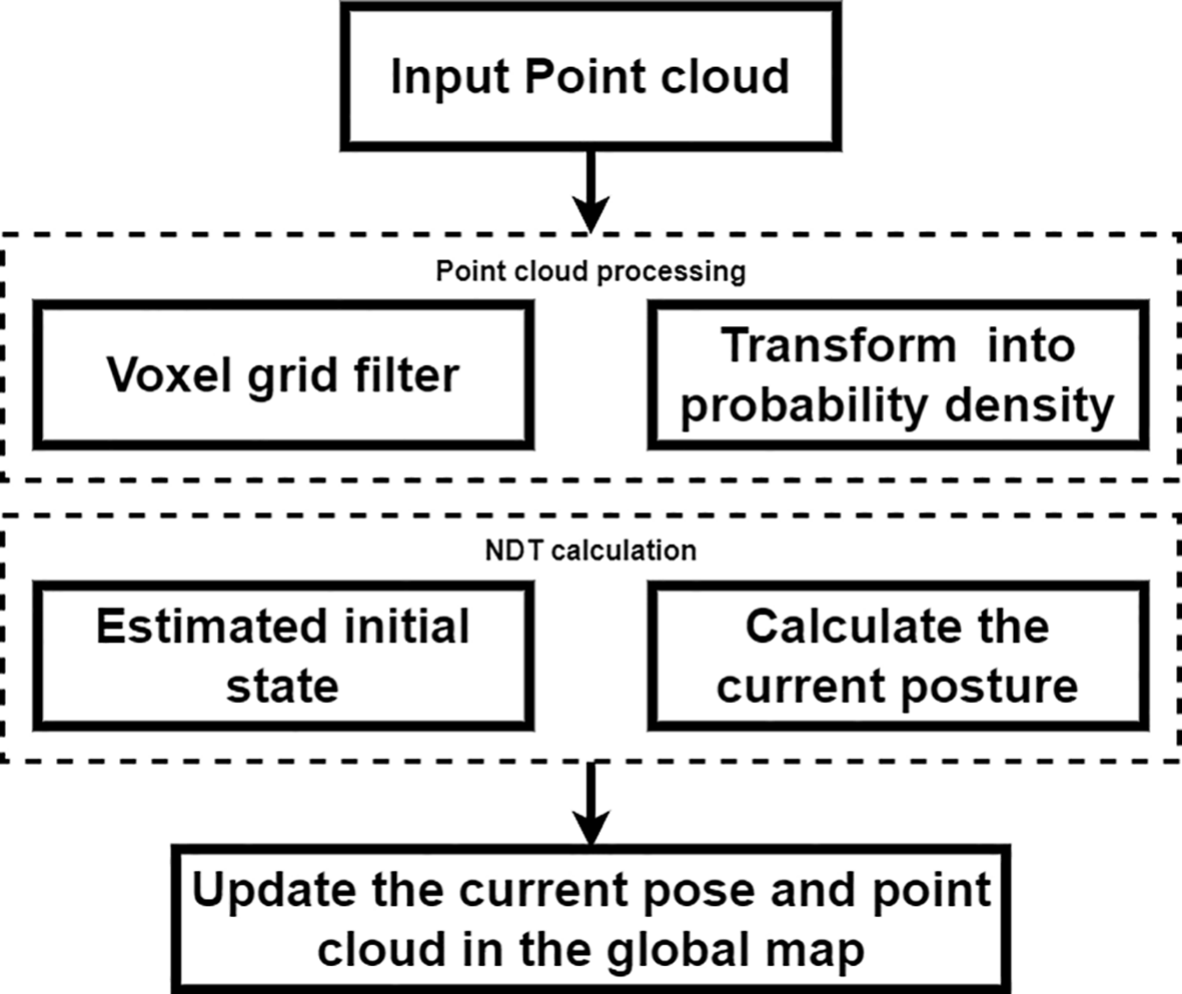
NDT mapping. NDT, normal distributions transform.

For Formula 4, the optimal solution was obtained by Newton’s optimization iteration as the best matching poses, and then the point cloud of each LiDAR scan frame was updated in the global map with the result of the matching poses. This process was repeated to overlay the point clouds to finally obtain the global map. The flowchart of the NDT mapping algorithm is shown in **Figure 3**.

There are several advantages to using the NDT method for environment construction in an orchard environment. First, it can adjust the map resolution and reduce the data volume by voxelizing the point cloud, which has a good downsampling effect on the information of all kinds of branches and leaves noise in the orchard. Second, the method has higher adaptability to subtle changes in the environment. When there is a change in the fruit trees within a divided voxel, the probability density description method can effectively reduce the error caused by subtle changes in the point cloud matching.

### 2.4 GNSS fusion NDT point cloud matching based on localization method

The NDT matching approach to localization is also based on the above-mentioned principle of great likelihood estimation for positional computation. By continuously comparing the input point cloud with the already recorded point cloud in each frame, the position corresponding to each frame is continuously output. However, this approach needs to give the initial value of the locus pose during the point cloud matching calculation, and the approximate solution of the locus pose is used to obtain the optimal result faster by subsequent Newtonian iterative optimization. If completely relying on NDT positioning, the system can only set the initial value of the positional pose once at the beginning, which will lead to positioning failure and no automatic correction. When the initial value is set incorrectly or the subsequent matching cannot obtain the result, the vehicle position falls into robot abduction. In order to solve this problem, the best method is to continuously provide the NDT with the initial position value and recalculate it through external data when the NDT fails. Considering that the orchard is outdoors, it is suitable to introduce GNSS information with high position positioning accuracy and convert the longitude and latitude data provided by GNSS into the initial position parameters of the vehicle and use them as iterative initial values for NDT position calculation, which changes the positioning method from single sensor work to multi-sensor fusion positioning.

**Figure 4:**
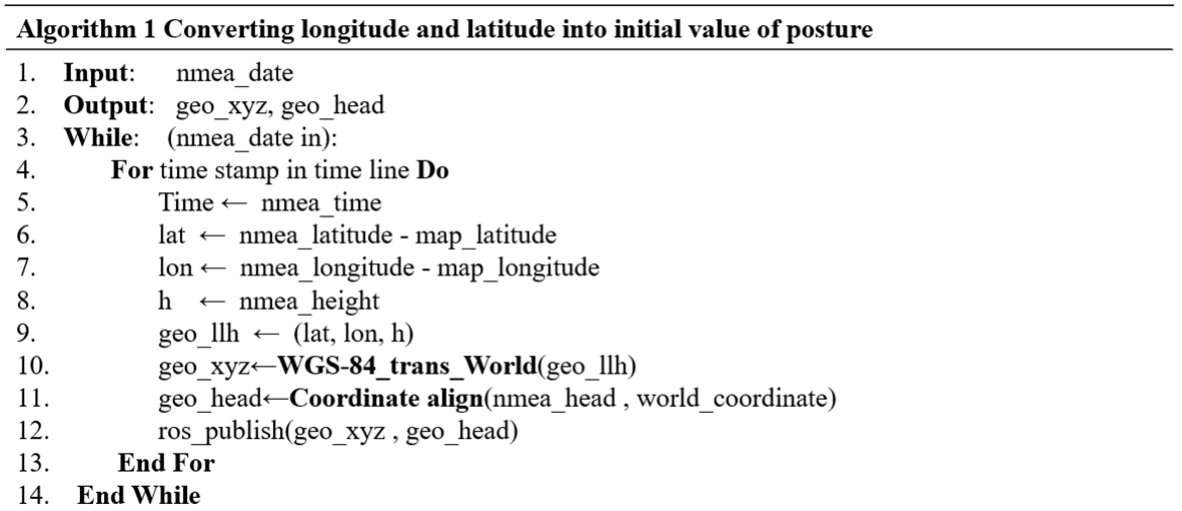
Pseudo code for conversion.

For the specific practice of converting RTK latitude and longitude information to vehicle position, this paper proposes a conversion procedure to convert it in real time, and its pseudo-code is shown in **Figure 4**. The program first records the latitude and longitude of the origin of the point cloud map and aligns the world coordinate system with it. Then, the relative latitude and longitude are obtained by subtracting the original world coordinate of the map from the input GNSS. Finally, the relative coordinate information is transformed into the map coordinate system in real time.

**Figure 5:**
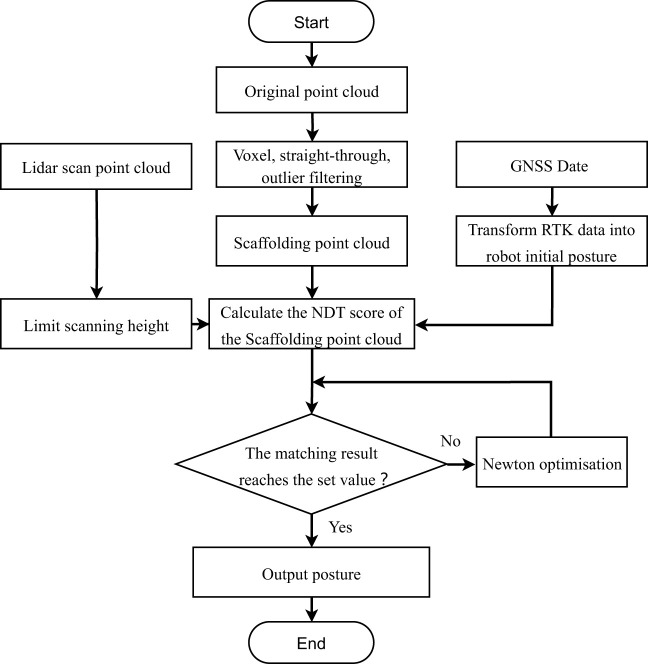
Flowchart of RTK fusion NDT location algorithm. RTK, real-time kinematics; NDT, normal distributions transform.

Considering the large variety of fruit trees, the timeliness and reliability of the point cloud generated by NDT mapping are poor, and the efficiency of the solution will be affected by a large amount of data in the subsequent point cloud matching. The trellis is a fixed and not easily deformed marker in the orchard, and it is the most ideal reference point cloud for alignment. The research uses the Point Cloud Library (PCL) to filter and extract it as the reference point cloud for NDT pose calculation. The specific flow of the point cloud processing and positioning method is shown in **Figure 5**. The input point cloud is first voxel filtered to reduce the overall data volume of the point cloud, then the lower height point cloud is cut in the form of straight pass filtering to retain the fruit tree and trellis point clouds, and finally, the redundant point clouds within the two trellises are cut by rows in the form of outlier filtering with straight pass filtering to retain the trellis point cloud alone. After the scaffold reference point cloud has been processed, the height of the input LiDAR point cloud is controlled to maximize the proportion of the input scaffold point cloud among all input point clouds, and the RTK-supplied positional information is used as the initial value for the NDT iterative algorithm to match the scaffold reference point cloud with the LiDAR input point cloud. The NDT first constructs a transformation matrix T containing six degrees of freedom according to the initial value of the pose and projects the input LiDAR point cloud into the reference point cloud coordinate system via the transformation matrix T. The algorithm calculates the matching score by comparing the position distribution of each cell of the input point cloud in the reference point cloud and superimposing the cells generated from the two sets of point clouds. When the matching score is less than the pre-defined matching threshold, the alignment parameter T is calculated by iterative Newtonian optimization to make the matching score meet the requirement, and the result is the position of the input point cloud. When the matching score does not meet the predetermined threshold, the positioning is judged to be lost. At this time, the positioning process is restarted, and the initial position information is recalculated from the previously obtained RTK coordinate information. The NDT calculation process is repeated to obtain the vehicle position information.

### 2.5 Vector map navigation path generation and tracing method

**Figure 6:**
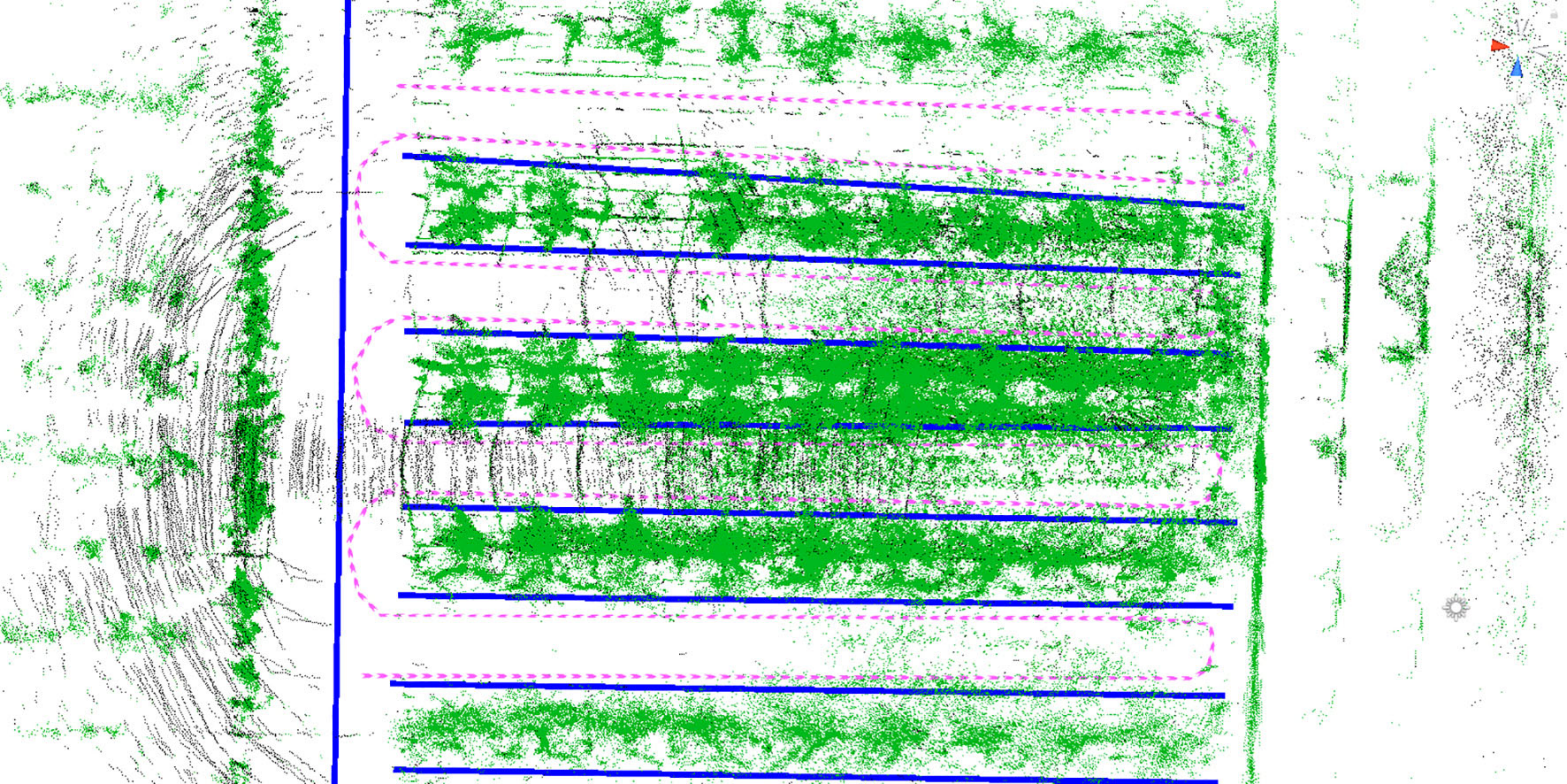
Vector map with manually labeled paths. The green part of the figure is the orchard point cloud, the pink route is the driving path, and the blue line is the driving boundary.

The task of mapping driveable paths in a 3D point cloud map is usually performed by means of high-precision vector maps in the field of autonomous driving. A vector map consists of point elements, lane elements, line elements, pole elements, surface elements, and so on. The lane information is generated by manually or automatically marking the position of each element in the point cloud. The complete vector map in the autonomous driving domain also has elements such as stop lines, traffic lights, and footpaths. Fruit trees are regularly arranged, which provides good conditions for map vectorization. Therefore, this paper uses a manually annotated vector map to plan the driving rules and only takes the four elements of the point node lane to form the basic elements of the navigation path. Each point element has its own ID number and coordinates position in a vector map, and the specific location of each point can be marked in the point cloud map. When a path is specified, the point element in the lane is converted into a node element. The node element defines the points on the driveable lane in the point cloud, and the location information of the lane elements can be defined by the node. The dtlane element is a point element that complements the lane shape information, including the directional angle between points and the curvature radius of the steering path. Lane elements are sequentially connected by node elements to build lanes and define the center of the driving route. These four elements are recorded in tabular form with their respective attributes and finally published separately in the point cloud map through the ROS system. Unity 3D Maptool plug-in is used to carry out manual vector map annotation in the orchard point cloud. The planned path is shown in **Figure 6**. The navigable path in the orchard is constructed by adding a point element and connecting each point to lane element. The same method is used to construct and transform road edges. The U-shaped line is taken as the fruit tree inter-row steering route, and the driving route map is built with three rows of fruit trees as the main object. Among them, the pink dotted line is the calibrated navigation route, the blue solid line is the prescribed form range, and the green part is the orchard point cloud. They are used to assist in labeling the driving line of a vector map to precisely locate the position of each element.

**Figure 7:**
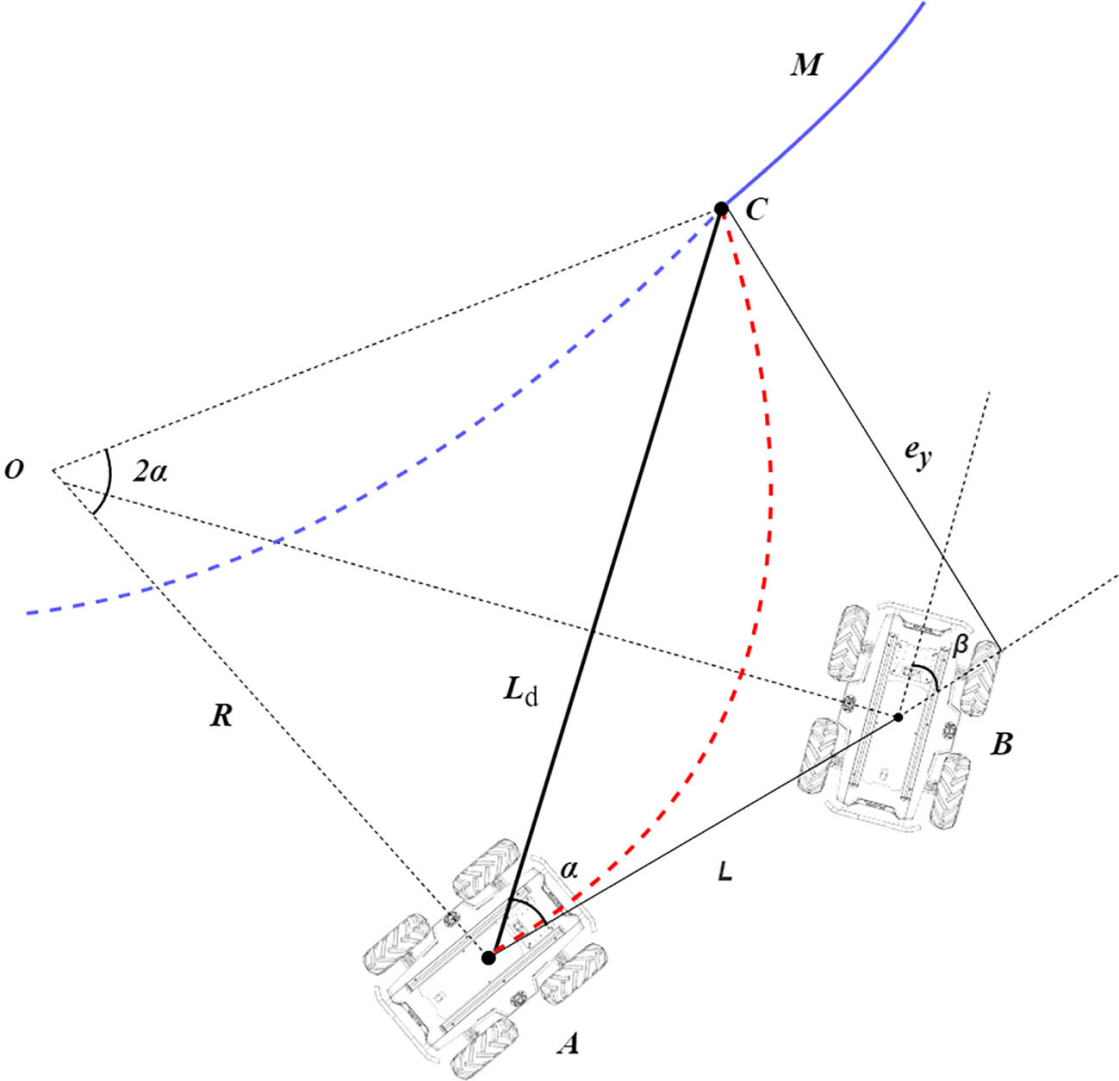
Pure pursuit model. Point *A* in the figure represents the current position, *C* represents the lookahead point position, and *B* represents the position of the vehicle during turning. The blue curve *M* represents the planned path, and the red curve *AC* represents the turning path. *α* is the heading angle at point *A*, *β* is the heading angle at point *B*, *L* is the distance from the center of the vehicle to the next position, *L_d_* is the chord of the steering curvature arc, and *e_y_* is the error in the lateral direction between the rear wheel center position and the lookahead point.

When the trajectory is generated, the final task of the navigation vehicle is to follow the trajectory. The planned trajectory in the orchard is relatively simple, and the mobile robot is a wire-controlled chassis satisfying the differential kinematic model, which can directly receive the linear and angular velocity commands sent from the ROS to achieve trajectory tracking. The kinematic model of the four-wheel chassis during tracing is modeled in **Figure 7**. Refer to pure path tracking (Coulter,1992). For the target trajectory *M*, a pre-sighting point *C* is selected on the reference path to be tracked, which is at a distance *L_d_* from the vehicle’s center of mass position, and the rear wheel center of the vehicle can be driven along a certain turning radius *R* to reach this point.

In triangle *OAC*, *AC* and *OC* satisfy the sine rule, and because angle *OCA* is complementary to angle *CAB*, Formula 5 can be obtained by using the sine rule.


(5) $$\frac{L_{d}}{\sin(2\alpha )}=\frac{R}{\sin(\frac{\pi }{2}-\alpha )}$$

where *L_d_* is the chord of the steering curvature arc, *α* is the heading angle at point *A*, and *R* is the radius of the steering curvature circle.

By simplifying Formula 5, the expression for the radius of curvature in terms of turning can be obtained, which is given by Formula 6.


(6) $$R=\frac{L_{d}}{2\sin\alpha }$$

In order to determine the heading angle at each position during tracking, assume that the vehicle travels from point *A* to point *B*. Because *β* is complementary to angle *OBA*, and angle *OBA* is complementary to angle *AOB*, the expression for the heading angle *β* can be obtained in triangle *AOB* using trigonometric relationships, which is given by Formula 7.


(7) $$\beta =\arctan\frac{L}{R}$$

where *β* is the heading angle and *L* is the distance from the center of the vehicle to the next position.

By substituting Formula 6 into Formula 7, a further simplified expression for the turning angle can be obtained, which is given by Formula 8.


(8) $$\beta =\arctan\frac{2L\sin\alpha }{L_{d}}$$

The lateral error *e_y_* is defined as the error in the lateral direction between the rear wheel center position and the lookahead point. In triangle *ABC*, the expression for the forward distance *L_d_* can be obtained using the sine rule, which is given by Formula 9.


(9) $$L_{d}=\frac{e_{y}}{\sin\alpha }$$

where *e_y_* is the error between the rear wheel center position and the pre-sighting point in the lateral direction.

By substituting Formula 9 into Formula 8, the final expression for the heading angle can be obtained, which is given by Formula 10.


(10) $$\beta =\arctan(\frac{2Le_{y}}{L_{d}{\;}^{2}})$$

During the motion at each moment, the turning angle is relatively small. Therefore, Formula 10 can be simplified to the form of Formula 11, which leads to the final heading angle control model.


(11) $$\beta =\frac{2L}{L_{d}{\;}^{2}}e_{y}$$

## 3 Results and discussions

### 3.1 NDT mapping trials

**Figure 8:**
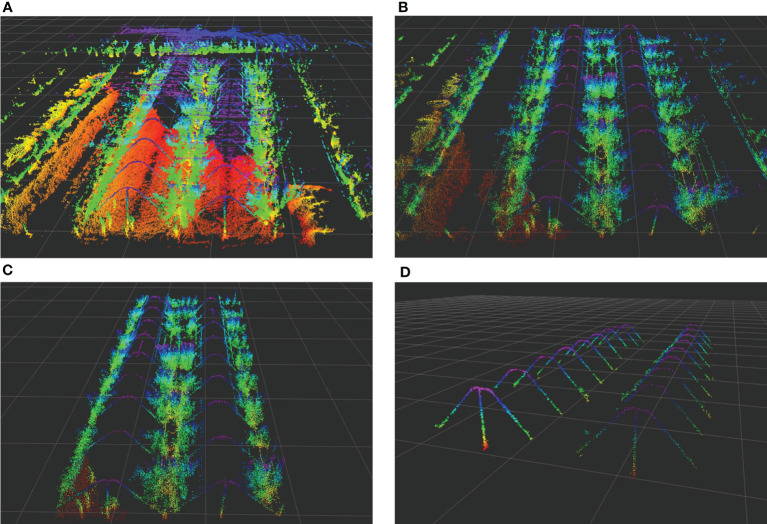
Orchard point cloud map processing method. **(A)** Original point cloud map. **(B)** Voxel filtering point cloud. **(C)** Pass-through filtering point cloud. **(D)** The trellis point cloud.

The NDT mapping test was conducted in a pear orchard. After connecting the hardware, the NDT mapping process was opened in ROS. The mobile platform was remotely controlled to move along the rows of trees and steered in a U-shaped trajectory at the end of each row. The final point cloud map of three rows of fruit trees is shown in **Figure 8A**. The point cloud of the orchard is very complex, it is unreliable to use the NDT-generated map as a reference map directly, and the original point cloud map needs to be processed using point cloud filtering to eliminate noise and compress map data. For the collected point clouds of Y-trellis orchards, the generated original point clouds were first retained 70% by downsampling to reduce the data volume. Then, the smaller dense point clouds were combined by voxel filtering and outlier filtering to reduce the overall resolution. The results are shown in **Figure 8B**, where the overall number of point clouds was effectively reduced. Three columns of fruit trees were selected as the test object, and the extra columns of fruit trees were removed by conditional direct-pass filtering, as in **Figure 8C**. Considering that the orchard has the characteristics of a trellis, it was chosen as a retained point cloud. In this way, the cluttered point cloud of the whole orchard can be preserved as a trellis point cloud, and it can be used as a high-precision map for positioning. The final processed localization point cloud is shown in **Figure 8D**, where the noisy and changeable canopy point clouds were filtered.

**Figure 9:**
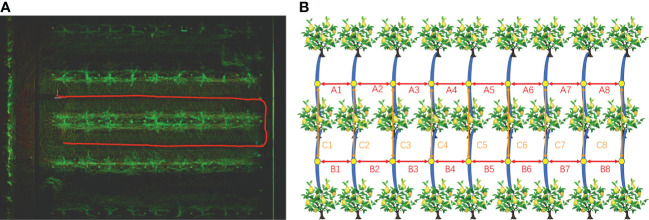
Orchard point cloud mapping test. **(A)** Initial orchard point cloud and data collection trajectory in RVIZ. **(B)** Mapping effect test schematic. A1 to A8 and B1 to B8 are the relative horizontal distances between the trellis, and C1 to C8 are the vertical relative distances between the vertical vertices.

After the trellis point clouds were processed, the relative position of each point cloud was measured in RVIZ using the measure plugin and compared with the real distance of the orchard to verify the accuracy of the mapping. **Figure 9A** shows the initial point cloud obtained in RVIZ, where the red curve is the movement trajectory of the vehicle. The mapping effect test method is shown in **Figure 9B**, where the trellis structure was divided into two rows. In an ideal situation, the horizontal distance between the trellis is 3 m, and the vertical distance between the vertical vertices is 6 m.

The results of the point cloud distance measurement tests in RVIZ are shown in **Table 2**. The maximum and minimum absolute errors of the point cloud in each row were 8 and 3 cm, respectively. The average measured value of the distance between rows was 3.02 cm, with an average standard deviation of 0.059 cm. The maximum and minimum absolute errors between reference points in each column were 11 and 8 cm, respectively. The average measurement of the inter-column distance was 5.975 cm with a standard deviation of 0.096 cm. The average error between rows and columns were within 3 cm, and the accuracy level meets the requirements of subsequent NDT matching localization. In order to understand the consistency of the point cloud measurements between rows and columns, the coefficient of variation (CV) was used to compare the three ABC data sets to verify the dispersion errors. The average CV of the three data sets was less than 2%, and the errors in both column and row directions were stable, which means that NDT mapping is reliable as a method for large-scale orchards. Compared to the application of extended Kalman filter (EKF)–SLAM in orchards (Wu, 2019), the accuracy level of NDT mapping is not much different from that of EKF–SLAM because this type of SLAM method determines the location of each point cloud by randomly scattering particles and calculating their distribution probability density, which is essentially similar to the probabilistic estimation method of NDT. However, NDT mapping uses 3D gridding to calculate probabilities, and the resulting orchard map was more informative and intuitive, which has the advantage of better map readability. Compared to the use of Cartographer in orchards (Xiong, 2021), this method has higher accuracy and is more advantageous in terms of mapping speed, as Cartographer needs to continuously optimize the already generated map, while this method only needs to overlay point clouds based on position.

**Table 2:** Reference point cloud relative distance measurement results.

No	Scaffold sampling point distance measurements (m)								Mean	Standard deviation	CV
	1	2	3	4	5	6	7	8			
Line A	A1	A2	A3	A4	A5	A6	A7	A8	3.015	0.064	0.021
	3.07	2.92	3.05	3.07	3.06	2.93	2.97	3.05			
Line B	B1	B2	B3	B4	B5	B6	B7	B8	3.026	0.054	0.018
	3.08	3.05	3.06	3.03	2.92	3.04	2.97	3.06			
Line C	C1	C2	C3	C4	C5	C6	C7	C8	5.975	0.096	0.016
	5.91	5.91	5.90	6.11	6.08	5.89	5.92	6.08			
Total											0.018

### 3.2 Fixed-point navigation test

**Figure 10:**
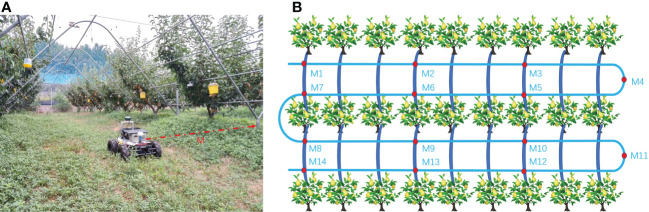
Fixed-point navigation tests. **(A)** Reference point distance measurement. **(B)** Schematic diagram of a fixed-point navigation test. M1 to M14 are measuring points on navigation path, and M is the distance between the tree column and the vehicle center.

**Figure 11:**
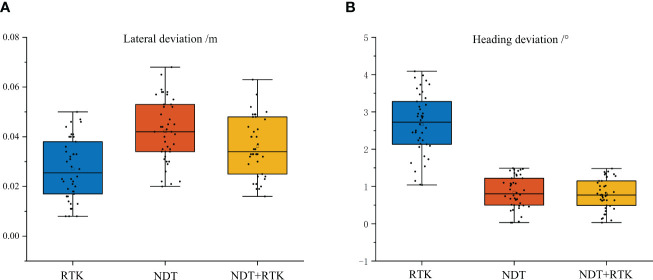
Fixed-point navigation test results. **(A)** Lateral deviation results. **(B)** Heading deviation results. The top and bottom horizontal lines are the maximum and minimum values, respectively. Three-quarters of the experimental error data points are in the box.

The fixed-point navigation test was conducted to verify whether the GNSS with NDT point cloud matching in the orchard could achieve the desired accuracy. The test site is shown in **Figure 10A**, and the specific test plan is shown in **Figure 10B**. The intersection points of navigation paths were selected, and the column-wise trellis was selected as the reference point. The measured distances between the bottom of the scaffold in the point cloud map and each reference point were taken as the true value 0 for localization. The vehicle was controlled to pass through points M1, M2, …, M14 in the order, and the distance to the reference point was measured when it reaches each point to obtain the lateral deviation. For heading positioning, the heading of the navigation track was taken as the true value 0. Since the path generated by the vector map was actually constructed in the form of points, the Angle between the two points adjacent to the measurement position and the due east direction was selected as the ideal course Angle, and the course deviation during its movement was recorded by the on-board IMU. The speed of the vehicle was set at 1.0 m/s and the lateral, heading deviations were recorded as absolute values. Meanwhile, in order to compare the effect of NDT point cloud matching positioning more clearly, three methods of GNSS positioning, NDT matching positioning, and GNSS fusion NDT positioning were selected for the above positioning tests. The lateral and heading errors of the three methods at 14 reference points were recorded, and the results of the box line plot are shown in **Figure 11**.

When the vehicle speed was 1.0 m/s, the average lateral deviation of RTK positioning was less than 3 cm, and the SD was less than 2 cm, while the average heading deviation was less than 3.5°, and the SD was less than 1°. Compared with that of the other two positioning methods, the lateral accuracy of RTK positioning was the highest and the data stability was strong, but compared with the point cloud matching-based positioning method, it was more susceptible to lateral and heading deviations due to obstruction in the orchard. The average lateral deviation of the NDT point cloud matching method was less than 5 cm, and the SD was less than 2 cm, while the average heading deviation was within 1°, and the SD was less than 0.6°. This positioning method was better in terms of lateral and heading accuracy, but during long-time operation, it can be interfered with by similar point cloud scenes and lead to positioning failure. It can be inferred that relying solely on NDT positional estimation as a navigation method is not stable. The GNSS fusion NDT positioning was comparable in accuracy performance to the NDT-only positioning method, with an average lateral deviation of less than 5 cm and an SD of less than 2 cm. The average heading deviation was within 1° and an SD of less than 0.6°, in accordance with its positioning theory. However, it is re-positioned by GNSS when NDT matching fails, and the method lasts longer in operation than pure RTK positioning. It can still rely on aligned trellis point clouds to maintain accurate positioning under some heavily shaded fruit tree canopies. It also provides a more stable heading position by means of point cloud matching and correcting deviations in the heading by numerous key point clouds, which can be more effective in tasks requiring high orientation accuracy. At the same time, the redundant positioning approach by combining two positioning information is more fault tolerant in real-life positioning tasks than relying on one sensor alone for positioning.

### 3.3 Navigation effect test

**Figure 12:**
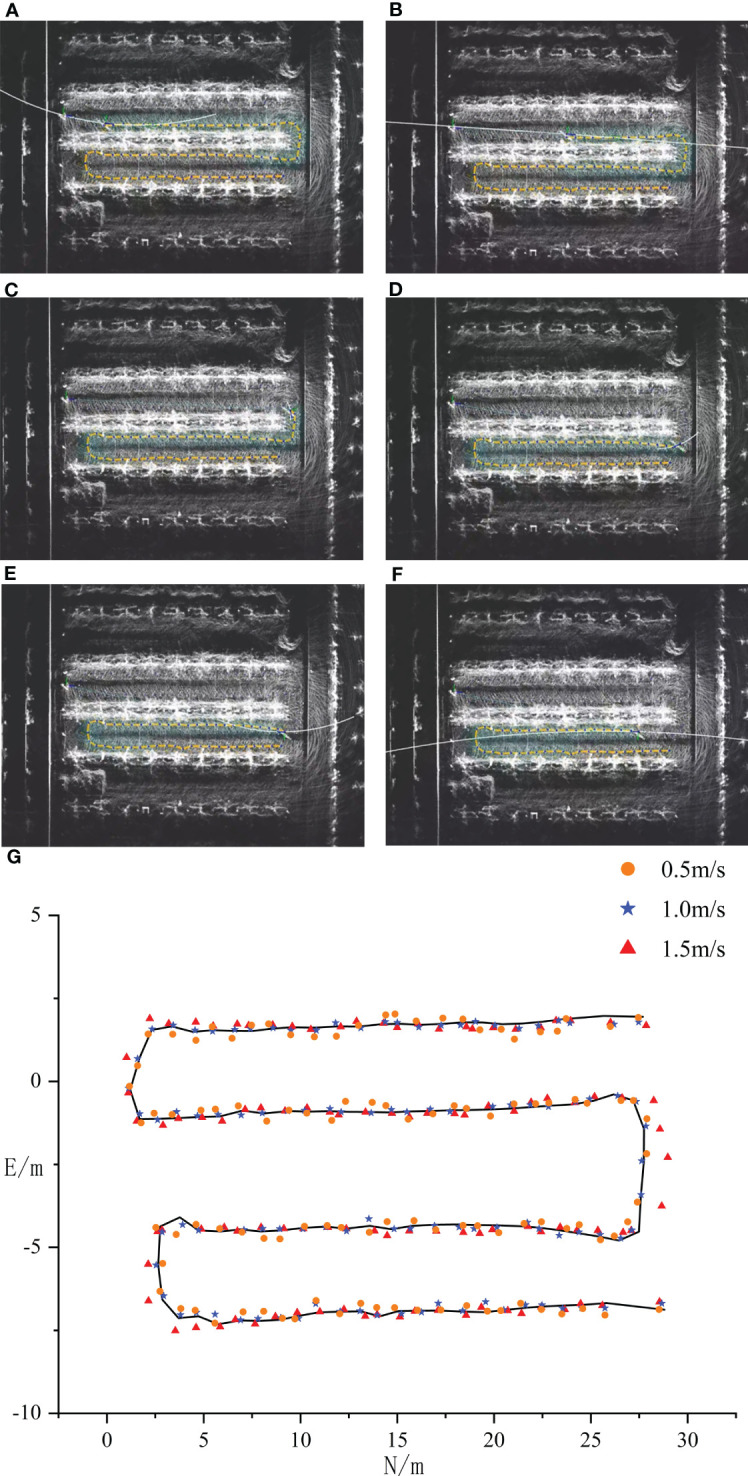
Navigation tests at different speeds. **(A–G)** The control line planning under straight and curved roads.

**Figures 12A–F** show different navigation positions when the speed was 1 m/s; the yellow path is the planning path, and the white line is the motion control line of pure path tracking planning. It was found that the planning line keeps changing with the path curvature, and the curvature was larger when navigating in a straight line and smaller when curving. Meanwhile, during the actual operation, the navigation effect changes significantly when different speeds were used for navigation. In order to explore the influence of different speeds on navigation accuracy, three driving speeds of 0.5, 1.0, and 1.5 m/s were selected, and the driving trajectories under each speed were recorded by RTK. The driving trajectories are shown in **Figure 12G**; the black curve is the planned path, and the round dots, stars, and triangular scatter points are the vehicle tracing paths under the three driving speeds. At the speed of 0.5 m/s, the maximum and minimum positioning errors of the tracing path were 13 and 3 cm, respectively, and the overall average error of the sampling points was 8 cm. At the speed of 1.0 m/s, the maximum and minimum errors of the tracing path were 6 and 1 cm, respectively, and the overall average error of the sampling points was 3.5 cm. At the speed of 1.5 m/s, the maximum and minimum errors of the tracing path were 15 and 5 cm, respectively, and the overall average error of the sampling point was 7 cm. When the speed was too slow, the heading adjustment of the navigation system was very frequent, and the tracing was incoherent. After it was combined with the pure path tracking, it can be found that the front view distance of the controller becomes smaller when the speed was too small, and the heading angle adjustment was large. At the same time, it can be found that the front view distance becomes longer when the speed was faster, and the tracking effect was stable in the straight trajectory, but the steering curvature was larger when passing through curves, resulting in a large radius through the curves. If the response lag of each sensor on the control platform was too fast, it will affect the navigation effect. When the vehicle was running at 1.0 m/s, the best tracing effect was achieved, as the speed setting affects the front view distance and thus changes the steering curvature. The best steering speed should be set according to the tree spacing. Based on the test, the best driving speed is 1.0 m/s, and the best forward-looking distance is 5.5 m.

## 4 Conclusion

Conducting various agronomic processes with an orchard autopilot platform is important for reducing human work time and improving operational accuracy in orchard production. Unlike scenarios in open environments such as field harvesting, the navigation system of an autonomous driving platform in an orchard environment is more difficult in terms of complex canopy handling and unstructured ground travel. In order to make the navigation in the orchard more suitable for continuous inter-row navigation and obstruction environment, this study used a four-wheel differential robot as the platform, constructed the environment using NDT Mapping’s 3D SLAM method, and processed it with the PCL for the Y-shaped trellis environment. At the same time, the problem of easy loss of positioning in complex scenes in orchards was solved by GNSS fusion NDT point cloud matching for positioning. The results showed that the accuracy of NDT mapping was 10 cm. The positioning accuracy reached 5 cm in the lateral direction and 1° in the aerial direction. During the practical tests, the method was able to perform continuous navigation in the orchard, but there were problems such as the large amount of point cloud processing, which caused the chassis motion control to stutter, and the controller forward-looking distance parameters could not be applied to different scale scenarios.

In future research, we plan to explore a point cloud localization method that is less computationally intensive to suit devices with lower computing power and reduce system latency. To address the issue of control algorithms, controllers with variable parameters or control methods such as Model Predictive Control (MPC) and Linear Quadratic Regulator (LQR) should be used to improve the stability of navigation control, enabling autonomous navigation of orchard vehicles in a cost-effective, efficient, and accurate manner.

## Data availability statement

The original contributions presented in the study are included in the article/**Supplementary Material**. Further inquiries can be directed to the corresponding authors.

## Author contributions

XLL and XHL conceived the ideas and provided project funding support. YX wrote the paper and conducted the tests and equipment debugging. XHL reviewed the paper and suggestions. YX and XHL performed the experiments and processed the data with the assistance of JP, LC, and ZZ. Assistance includes moving equipment, recording test data, and preparing test materials. All authors contributed to the article and approved the submitted version.
